# Clinical characteristics and visual outcomes of work-related open globe injuries in Japanese patients

**DOI:** 10.1038/s41598-020-57568-9

**Published:** 2020-01-27

**Authors:** Shohei Morikawa, Fumiki Okamoto, Yoshifumi Okamoto, Yoshinori Mitamura, Hiroto Ishikawa, Kozo Harimoto, Tetsuo Ueda, Taiji Sakamoto, Kazuhiko Sugitani, Osamu Sawada, Junya Mori, Yoshihiro Takamura, Tetsuro Oshika, Japan-Clinical Research of Study (J-CREST) group, Naoki Inomoto, Fumi Gomi, Masaru Takeuchi, Nahoko Ogata, Toshifumi Yamashita, Hiroki Otsuka, Seiji Sameshima, Hideki Shiihara, Yoshio Hirano, Tsutomu Yasukawa, Masahito Ohji, Takamasa Kinoshita

**Affiliations:** 1 Department of Ophthalmology, Faculty of Medicine, University of Tsukuba, Tsukuba, Ibaraki, Japan; 2 Department of Ophthalmology, Institute of Health Biosciences, The University of Tokushima Graduate School, Tokushima, Japan; 3 Department of Ophthalmology, Hyogo College of Medicine, Nishinomiya, Hyogo, Japan; 4 Department of Ophthalmology, National Defense Medical College, Saitama, Japan; 5 Department of Ophthalmology, Nara Medical University, Kashihara, Nara, Japan; 6 Department of Ophthalmology Kagoshima University Graduate School of Medical and Dental Sciences, Kagoshima, Japan; 7 Department of Ophthalmology and Visual Science Nagoya City University Graduate School of Medical Sciences, Nagoya, Japan; 8 Department of Ophthalmology, Shiga University of Medical Science, Otsu, Shiga, Japan; 9 Department of Ophthalmology, Sapporo City General Hospital, Sapporo, Hokkaido, Japan; 10 Department of Ophthalmology, Faculty of Medical Sciences, University of Fukui, Fukui, Japan

**Keywords:** Diseases, Risk factors, Clinical trial design

## Abstract

Purpose: To investigate the clinical characteristics and visual outcomes of patients with work-related open globe injuries (OGIs) and compare them with patients with non-work-related OGIs. Design: Retrospective, observational, multicentre, case-control study. Methods: A total of 374 patients with work-related OGIs and 170 patients with non-work-related OGIs who presented to hospitals that belong to the Japan-Clinical Research of Study group from 2005 to 2015 were included in this study. Clinical data including age, sex, initial and final visual acuity, type of open globe injury, lens status, zone of injury, wound length, and presence of proliferative vitreoretinopathy, retinal detachment, expulsive haemorrhage, and endophthalmitis were recorded. Main Outcome Measures: Visual acuity. Results Work-related OGIs were associated with younger age, male sex, better initial and final visual acuity, more laceration, smaller wounds, presence of retinal detachment, and expulsive haemorrhage, compared with non-work-related OGIs. Multiple regression analysis revealed that final visual acuity is significantly associated with initial visual acuity, wound length, and the presence of proliferative vitreoretinopathy in work-related OGIs. Conclusions: Work-related OGIs showed better visual outcomes than other OGIs. Initial visual acuity, wound length, and the presence of proliferative vitreoretinopathy are predictors of visual outcomes in patients with work-related OGIs.

## Introduction

Ocular trauma is a significant problem commonly encountered in healthcare settings throughout the world. It is estimated that 55 million eye injuries occur around the world each year^1^. The incidence of OGI cases that need hospitalization ranges from 3.0–51.0 per 100,000 population^2–10^.

Open globe injuries (OGIs), defined as full thickness wounds of the eye wall, are an important cause of blindness and visual morbidity^11^, with 203,000 OGI cases occurring each year around the world, and 1.2–6.0 per 100,000 cases requiring medical care^2–10,12^. Common mechanisms of OGIs include falls, occupational accidents, sports, traffic accidents, fishing, and assaults^1,13–16^. Previous studies reported that work-related OGIs accounted for 28.3% of all OGI cases^17^ and 79.1% of the patients were young males^18^.

It has been reported that the rates of intraocular foreign body and traumatic cataract are high in work-related OGIs and poor initial visual acuity and zone of injury are known factors associated with a poor visual acuity prognosis^11,19^.

However, there is presently no report that focused solely on work-related OGIs, or compared work-related OGIs with non-work-related OGIs in Japan. In the present study, we investigated the clinical characteristics and visual outcomes of patients with work-related OGIs, and evaluated the difference between work-related and non-work-related OGIs.

## Results

A total of 374 patients with OGIs were enrolled in this study. Table 1 shows the characteristics of patients with work-related OGIs and those of patients with non-work-related OGIs. Out all the recorded injuries, 170 (45.5%) were work-related OGIs and 204 (54.5%) were non-work-related OGIs. The patients with work-related OGIs, consisted of 161 males (94.7%) and 9 females (5.3%), with an average age of 49.9 ± 17.2 years (range: 18–88 years). For the non-work-related OGI group, which comprised 114 males (55.9%) and 90 females (44.1%), the average age of the patients was 62.6 ± 24.0 years (range: 2–101 years).

**Table 1:** Comparisons of various parameters between work-related open globe injuries and non-work-related open globe injuries.

Parameters	Work group	Non-work group	P value
Age (years)	49.9 ± 17.2	62.6 ± 24.0	<0.001*
Gender (men/women)	161/9	114/90	<0.001*
Initial visual acuity (logMAR)	1.7 ± 1.1	2.6 ± 0.5	<0.001*
Final visual acuity (logMAR)	0.8 ± 1.1	1.8 ± 1.1	<0.001*
Type of open globe injury (rupture or laceration)	46/124	159/45	<0.001*
Wound length (mm)	4.9 ± 4.6	9.8 ± 6.3	<0.001*
Presence of PVR (+/−)	11/121	13/131	0.84
Presence of RD (+/−)	39/127	93/93	<0.001*
Presence of EH (+/−)	13/115	39/101	<0.001*
Presence of EO (+/−)	12/158	7/191	0.13

Values are presented as the mean ± standard deviation. *Significant correlation between the parameters (Student’s t-test and chi-square test).

Work group = work-related open globe injuries; Non-work group = non-work-related open globe injuries.

LogMAR: logarithm of the minimum angle of resolution; RD: retinal detachment; PVR: proliferative vitreoretinopathy; EH: expulsive hemorrhage; EO: endophthalmitis.

Of the 170 patients in the work-related OGI group, 46 patients (27.1%) presented with rupture and 124 (72.9%) with laceration. Regarding lens status, 141 eyes were phakic, nine eyes were pseudophakic, 11 eyes were aphakic, and the remaining nine eyes were unclear. Regarding zone of injuries, 93 eyes were categorized as zone I, 37 eyes as zone II, 36 eyes as zone III, and the remaining four eyes were unclear. PVR was observed in 11 eyes (8.3%), RD in 39 eyes (23.5%), EH in 13 eyes (10.1%), and EO in 12 eyes (7.1%). About treatment 62 eyes were only sutured a wound, 14 eyes were received cataract surgery, 86 eyes were received vitreous surgery, three eyes received enucleation, and the remaining five eyes were unclear. One hundred eight eyes underwent first surgery and the other 62 eyes underwent second surgery. The average of period from the injury to the medical institution was 1.6 days and there was no regional difference.

Out of the 204 patients in the non-work-related OGI group, 159 patients (77.9%) presented with rupture and 45 (22.1%) with laceration. Regarding lens status, 98 eyes were phakic, 33 eyes were pseudophakic, and the other 55 eyes were aphakic. Regarding zone of injuries, 53 eyes were categorized as zone I, 51 eyes as zone II, and the other 88 eyes as zone III. PVR was observed in 13 eyes (9.0%), RD in 93 eyes (50.0%), EH in 39 eyes (27.9%), and EO in seven eyes (3.5%).

Initial visual acuity was 1.7 and final visual acuity was 0.8 in the work-related OGI group, indicating that the visual acuity was significantly improved by surgery (p < 0.001). In the non-work-related OGI group, visual acuity was also significantly improved by surgery (Initial visual acuity was 2.6; final visual acuity was 1.8, p < 0.001).

The patients in the work-related OGI group were significantly younger, tended to be male, and had a higher incidence of laceration, RD, and EH than those in the non-work-related OGI group. The initial visual acuities and final visual acuities of patients in the work-related OGI group were better than those of patients in the non-work-related OGI group.

In the work-related OGI group, final visual acuity was significantly correlated with initial visual acuity and wound length, and had a significant association with the type of injury, presence of PVR, RD, and EH (Table 2). There was no significant difference in final visual acuity between institutions (p = 0.06). Results of a multiple regression analysis showed that patients’ final visual acuity was significantly associated with their initial visual acuity (p < 0.001), wound length (p < 0.001), and the presence of PVR at first visit to hospital (p < 0.001).

**Table 2:** Relationship between final visual acuity and preoperative parameters in work-related open globe injuries.

Preoperative parameters	Final visual acuity	
	r	p
Age (years)	0.058	0.44
Gender	—	0.14
Initial visual acuity (logMAR)	0.58	<0.001^*^
Type of open globe injury (rupture or laceration)	—	<0.001^*^
Wound length (mm)	0.52	<0.001^*^
Presence of PVR (+/−)	—	<0.001^*^
Presence of RD (+/−)	—	0.11
Presence of EH (+/−)	—	0.04
Presence of EO (+/−)	—	0.74

^*^Significant correlation between the parameters (Spearman’s rank correlation coefficient and Student’s t-test).

LogMAR: logarithm of the minimum angle of resolution; RD: retinal detachment; PVR: proliferative vitreoretinopathy; EH: expulsive hemorrhage; EO: endophthalmitis.

## Discussion

We extracted work-related OGI cases from a large number of OGI cases registered in the J-CREST database, investigated the characteristics of the cases, and compared them with those of OGIs from other causes. A prior study reported that the average age of patients who sustained work-related OGIs was 35.8 years^20^; some other previous studies reported that majority of patients with work-related OGIs are male (79–98%)^18–20^. In the present study, the average age of patients in the work-related OGI group was 49.9 years and 94.7% were male, which was similar to the findings of other studies.

Bauza *et al*. found a significant difference in the types and zones of injuries, initial and final visual acuity, enucleation rate, incidence of cataracts, hyphema, RD, EH, and afferent pupillary defect between work-related OGIs and non-work-related OGIs^19^. Kanoff *et al*. reported that initial and final visual acuity was better for patients with work-related OGIs than for those with non-work-related OGIs^20^. We found a significant difference in age, sex, initial and final visual acuity, types of injury, wound length, and the presence of RD and EH between the two groups.

In the present study, patients who sustained work-related OGIs were relatively younger, tended to be male, and had a higher incidence of laceration than those in the non-work-related OGI group. This is probably because OGIs occur relatively frequently after a fall among the elderly. In this study, 59% of non-work-related OGIs were caused by falls. Previous studies reported that 14–32% of patients with OGIs sustained their injuries by falling^21,22^. In geriatric populations_,_ the most common mechanism of open globe injuries is falls (65%)^23^, and women were main victims of fall-related OGIs; the mean age of OGI patients in this demographic is 65.8–80.6 years old^21,24^. Further, 90.8% of the patients who accidentally fell presented with ruptures^21^. Our results are consistent with the findings of the preceding studies.

The initial visual acuity and final visual acuity of patients in the work-related OGI group were superior to those of the patients in the non-work-related OGI group. The probable cause was that the incidence of laceration was higher in the non-work-related OGI group than in the work-related OGI group. The initial and final visual acuity of rupture patients in our study was poorer than that of patients with laceration; this result is a generally well-recognised finding^24–26^.

Our multiple regression analysis showed that in the work-related OGI group, initial visual acuity, wound length, and the presence of PVR were associated with final visual acuity. A number of previous reports revealed that poor initial visual acuity is a poor prognostic factor in OGIs, fall-related OGIs, and work-related OGIs^19,21,24,25,27–30^. In addition, a large wound^30^ and PVR^24^ are associated with final visual acuity in OGI cases. These findings were consistent with our results.

This study has some limitations. This was a retrospective study, and there were some missing data such as lens state, zone and treatment. In addition, the follow-up period was relatively short. Future studies with a longer follow-up period are needed to confirm and validate the findings of this study.

In summary, we assessed work-related OGI cases in a multicentre study in Japan. Patients with work-related OGIs were younger and exhibited better visual outcomes than those with non-work-related OGIs. Initial visual acuity, wound length, and the presence of PVR were found to be associated with the visual outcomes of work-related OGIs.

## Methods

This was a retrospective, comparative, observational, case-control study of patients who had been diagnosed with an OGI and followed up for at least six months at ten core hospitals that belong to the Japan Clinical Retina Study group (i.e., University of Tsukuba, Tokushima University Graduate School, Hyogo College of Medicine, National Defense Medical College, Nara Medical University, Kagoshima University Graduate School of Medical and Dental Sciences, Nagoya City University Graduate School of Medical Sciences, Shiga University of Medical Science, Sapporo City General Hospital, and Faculty of Medical Sciences University of Fukui) from January 1, 2005 to January 31, 2015. The Japan Clinical Retina Study group was formed for the purpose of analysing disease statistics and pathological conditions by accumulating and sharing data among multiple centres across Japan. We conducted this study in accordance with the tenets of the Declaration of Helsinki, and received approval from the institutional review boards of each hospital. Informed consent was obtained from all individual participants included in the study.

The obtained data were classified into two categories (work-related or non-work-related) depending on the mechanisms of the OGIs of the patients. Clinical data, including patients’ age, sex, initial visual acuity, final visual acuity, the type of injury (i.e., rupture or laceration), lens state, zone of injury, wound length, presence of proliferative vitreoretinopathy (PVR), retinal detachment (RD), expulsive haemorrhage (EH), and endophthalmitis (EO) at the first visit to hospital, were collected. In OGIs, zone I injuries are confined to the cornea and limbus, zone II injuries involve the anterior 5 mm of the sclera, and zone III injuries involve full-thickness scleral defects >5 mm posterior to the limbus^11^.

Patient visual acuity was converted from decimal to logarithm of the minimum angle of resolution (logMAR) values. Visual acuity of counting fingers, hand motion, light perception, and no light perception were assigned logMAR values of 1.85, 2.3, 2.8, and 2.9, respectively^31,32^. Mean scores and standard deviations were calculated for patient age, visual acuity, and wound length.

Relationships between final visual acuity and patient age, initial visual acuity, and wound length were examined using the Spearman’s rank correlation coefficient. The unpaired t-test was used to compare final visual acuity with parameters such as sex, type of injury (rupture or laceration), and the presence of PVR, RD, EH, and EO. The paired t-test was used to compare initial visual acuity with final visual acuity in each group; the test was also used to compare age, initial and final visual acuity, and wound length between the two groups. The chi-square test was performed to compare sex, the type of injury (rupture or laceration), and the presence of PVR, RD, EH, and EO between the two groups. The difference in final visual acuity between institutions were compared with Kruskal-Wallis test. Variables trending to statistical significance in the univariate analysis (P < 0.05) were included in the multiple regression analysis. All tests of association were considered statistically significant if p < 0.05. The analyses were carried out using StatView (version 5.0, SAS Inc., Cary, NC, USA).

## Data Availability

The datasets generated during and/or analysed during the current study are not publicly available.
